# TRAK App Suite: A Web-Based Intervention for Delivering Standard Care for the Rehabilitation of Knee Conditions

**DOI:** 10.2196/resprot.4091

**Published:** 2015-10-16

**Authors:** Irena Spasić, Kate Button, Anna Divoli, Satyam Gupta, Tamas Pataky, Diego Pizzocaro, Alun Preece, Robert van Deursen, Chris Wilson

**Affiliations:** ^1^ School of Computer Science & Informatics Cardiff University Cardiff United Kingdom; ^2^ School of Healthcare Sciences Cardiff University Cardiff United Kingdom; ^3^ Cardiff and Vale University Health Board National Health Service Cardiff United Kingdom; ^4^ Pingar Auckland New Zealand

**Keywords:** internet, social media, web applications, mobile applications, usability testing, knee, rehabilitation, exercise, self-management

## Abstract

**Background:**

Standard care for the rehabilitation of knee conditions involves exercise programs and information provision. Current methods of rehabilitation delivery struggle to keep up with large volumes of patients and the length of treatment required to maximize the recovery. Therefore, the development of novel interventions to support self-management is strongly recommended. Such interventions need to include information provision, goal setting, monitoring, feedback, and support groups, but the most effective methods of their delivery are poorly understood. The Internet provides a medium for intervention delivery with considerable potential for meeting these needs.

**Objective:**

The objective of this study was to demonstrate the feasibility of a Web-based app and to conduct a preliminary review of its practicability as part of a complex medical intervention in the rehabilitation of knee disorders. This paper describes the development, implementation, and usability of such an app.

**Methods:**

An interdisciplinary team of health care professionals and researchers, computer scientists, and app developers developed the TRAK app suite. The key functionality of the app includes information provision, a three-step exercise program based on a standard care for the rehabilitation of knee conditions, self-monitoring with visual feedback, and a virtual support group. There were two types of stakeholders (patients and physiotherapists) that were recruited for the usability study. The usability questionnaire was used to collect both qualitative and quantitative information on computer and Internet usage, task completion, and subjective user preferences.

**Results:**

A total of 16 patients and 15 physiotherapists participated in the usability study. Based on the System Usability Scale, the TRAK app has higher perceived usability than 70% of systems. Both patients and physiotherapists agreed that the given Web-based approach would facilitate communication, provide information, help recall information, improve understanding, enable exercise progression, and support self-management in general. The Web app was found to be easy to use and user satisfaction was very high. The TRAK app suite can be accessed at http://apps.facebook.com/kneetrak/.

**Conclusions:**

The usability study suggests that a Web-based intervention is feasible and acceptable in supporting self-management of knee conditions.

##  Introduction

### Management of Knee Conditions

Musculoskeletal conditions are the second largest contributor to years lived with disability [1]. In the United Kingdom, a total of 33% of individuals aged 45 and over have sought treatment for osteoarthritis, with the knee being the most commonly affected joint [2]. The incidence of acute knee injuries is reported to be at a rate of 2.29 per 1000 in US population [3]. In the Netherlands, 45-55% of acute knee injuries develop into a long-term medical condition [4]. Patients may still experience movement deficiency 1 year following knee surgery [5]. In particular, participation restrictions may persist 2 years following total knee replacement [6].

To support the recovery or management of a long-term musculoskeletal condition, physiotherapy rehabilitation is typically recommended. Standard care for the rehabilitation of knee conditions involves exercise programs and information provision [7,8]. However, current methods of rehabilitation delivery struggle to keep up with large volumes of patients and the length of treatment required to maximize the recovery. Therefore, the development of novel interventions to support self-management is strongly recommended. Such interventions need to include information provision, goal setting, monitoring, feedback, and support groups, but the most effective methods of their delivery are poorly understood and require further research [9,10]. Finally, treatments need to be personalized, that is, targeted at individual needs, to improve prospects of rehabilitation [10].

### Web-Based Intervention Approaches

Some argue that the Web-based approaches are more accessible, more convenient for patients, and can help counteract the current shortage of skilled therapists [11]. They can also be more effective in acquiring declarative knowledge [12]. Focusing on therapies that addressed deficiencies in patient knowledge and self-management skills, Web-based approaches were found to be more effective in increasing participation and exercise time [13]. In the context of physical activity interventions, it was found that over half of the controlled trials of Web-based interventions reported positive behavioral outcomes [14]. Similarly, findings of a systematic review demonstrated the effectiveness of Web-based patient education interventions, that is - interventions that are associated with producing changes in self-care behavior, on patient outcomes [15]. A meta-analysis of Web-based cognitive behavioral interventions demonstrated a small effect when using pain scale as the main outcome in comparison to waiting list control groups [16]. These results indicate the potential of Web-based interventions as a therapeutic tool for chronic pain associated with decreasing treatment costs and side effects. In general, Web-based interventions in patients with somatic diseases were found to be effective/cost effective, or at least promising in this regard [17]. In particular, Web-based physical activity interventions proved to be more effective than a waiting-list strategy [18].

Although Web-based behavioral interventions can be effective, poor adherence is commonly associated with such interventions [19]. The differences in technology and interaction, rather than the health care area itself, were found to be good predictors of adherence. The most effective Web-based interventions are interactive and flexible, thereby allowing patients to select information that is of relevance to them, and their learning at a particular point in time [15]. In addition, social media offer an opportunity to improve the efficiency and effectiveness of health care by providing an alternative mechanism to facilitate self-management of chronic diseases [20].

### The Health Care Industry and the Web

“Social media” is a term used to refer to a group of Web-based apps that allow the creation and exchange of user-generated content [21,22], such as social networks, forums, blogs. To reach stakeholders, leverage collaboration, and personalize care, the potential of social media needs to be explored [23-25]. They have already demonstrated a rising influence on patients' decision-making process (eg, what services to seek out, which doctor to consult). Not surprisingly, it has been suggested that social media will inevitably be an integral part of the future landscape of health care [26]. However, the accent here is on the word "future," because the health care industry is currently lagging behind in its use of social media to communicate with patients. For example, a study of social media use among Fortune 100 companies revealed that health care was the least active industry in this area [27]. Similarly slow adoption rates have been reported in the United Kingdom [28]. This tendency may be explained by a combination of various factors.

### Health Care Industry Factors for Web Resistance

Publicly financed health care organizations such as the National Health Service (NHS) have traditionally been risk averse. Subject to budgetary constraints, they strive to minimize the possibility of failure. Therefore, they tend to only adopt widely accepted, proven technologies [29].

There is a lack of best practices and robust metrics to measure success of investments in social media and produce reliable return-on-investment figures. Although there are several surveys, case studies, trials, and working examples, systematic evidence on the clinical outcomes of social-media-enabled health interventions is yet insufficient [23].

There is a general lack of trust. Social media are rife with misinformation. In heavily standardized and regulatory-driven industries such as health care, it is imperative to provide well-recognized and accredited sources of information [30].

Privacy, security, confidentiality, and liability questions present further concerns [25]. Communication between health care providers and patients must comply with current data security and privacy legislation [31,32]. Because of increased privacy laws and regulation, health care-centric discussions need greater moderation than discussions related to any other industry.

Health professionals tend to link the use of social media to deprofessionalization of their expertise, and they believe that it can undermine the traditional doctor-patient relationship [33].

Clearly, there are numerous hurdles to overcome, and engaging with social media can incur considerable risks. Despite their increasing popularity, their uncontrolled nature raises serious concerns about the quality, reliability, and accuracy of health information. The benefits health care may gain from investing into this relatively new technology are still fairly unclear [34]. Nonetheless, it provides a great opportunity to improve the quality of communication between patients and health care professionals, thus potentially improving health outcomes. In particular, the emergence of social networking sites (SNSs) created entirely new ways of social interactions. The traditional vertical dissemination has been replaced with a horizontal flow of information. Social networks dedicated to patients function as virtual support groups, which can offer a wide range of information and support needs that may not be met as part of conventional health care [35]. For example, the website “PatientsLikeMe” [36] was designed to facilitate conversation between patients suffering from the same medical condition. Another example is CureTogether [37], which helps its users to anonymously track the progress of conditions, collect data to aid research, and help patients better understand their bodies.

The majority of Web-based physical activity interventions focused on health promotion [38,39], often in a specific medical context such as obesity [40], diabetes, [41], or aging [42], but very few exist in areas such as osteoarthritis [43] or rheumatoid arthritis [44], where physical therapy is recommended. To our best knowledge, there is no evidence of a successful interactive app for patients with acute knee conditions.

##  Methods

### Primary Aim of the Study

The primary aim of this study was to demonstrate the feasibility of a Web-based, interactive app, and to conduct a preliminary review of its practicability as part of a complex medical intervention in the rehabilitation of knee disorders. Based on the precognitions of stakeholder expectations, our objective was to examine possible ways of facilitating remote self-care. Ethical approval for this study has been granted from the National Research Ethics Service via the South East Wales Research Ethics Committee Panel (ref 10/MRE0928).

### Choosing the Platform

Incorporating the social networking aspect into the proposed app is a critical success factor. There are 2 ways to accomplish this: (1) by organizing a new community from scratch or (2) carving out a chunk from the audience of the mainstream SNSs. Open source social networking platforms such as Elgg [45], Oxwall [46], or BuddyPress [47] provide an out-of-the-box solution for building new online societies. A disadvantage of this approach is that users need to register and remember their login credentials to yet another account and learn how to effectively use a new software environment. Alternatively, Facebook Login [48], Twitter OAuth [49], Yahoo OAuth [50], Google OAuth 2.0 [51], and Disqus [52] offer ways to integrate third-party websites with social media by allowing authentication and content authoring from external websites. Additionally, Facebook allows developers to host their apps on Facebook, which allows effortless promotion via “likes” and recommendations as well as contextual advertising. A wealth of user information (name, age, interests, groups, friends, etc) comes for free and users are faced with a familiar interface, which requires no special training, thus, not incurring a significant learning overhead. A Facebook app requires no installation, is platform neutral in terms of operating systems and Web browsers, and is, in principle, accessible from any Internet-enabled device. The interactive nature of Facebook and communication with other users makes it straightforward to integrate a virtual support group into the app. However, such communication together with the collection of user information may raise privacy concerns. This means that, in addition to Facebook's own privacy policy [53], it needs to be made clear to the users how their information will be used within the app. From developers' perspective, the negative consequences of choosing Facebook as a platform include loosing independence and developing to interfaces that may change, possibly breaking the app.

In the context of self-management interventions, Facebook seems like a suitable platform due to its widespread usage. In the United Kingdom, 61.1% of Internet users use Facebook at least monthly [54]. Similarly, in the United States, 74% of adult Internet users use SNSs, with Facebook being the preferred choice (71% of adult users) [55]. The percentage of Internet users within a specific age group who use Facebook is high in both young and middle-aged adults: 86% of users are in the age group of 18-29 years, 73% in the age group of 30-49 years, and 57% in the age group of 50-64 years [56]. Overall, Facebook users in the United States are currently distributed across different age groups as follows: 13-17 years (5.4%), 18-24 years (23.3%), 25-34 years (24.4%), 35-54 years (31.1%), and over 55 years (15.6%), with the 3 older groups recording a growth of 32.6%, 41.4%, and 80.4%, respectively, within the last 3 years [57]. This trend is contrary to popular belief that Facebook is mostly used by teenagers. When it comes to gender, the distribution of Facebook users is fairly balanced in comparison to other SNSs; there are slightly more female (53.3%) than male (45.6%) users. On the other side, acute knee injuries are most prevalent in young and middle-age adults (75.6% of knee injuries occur in men with a mean age of 32.9 years, compared with 24.4% in women with a mean age of 35.3 years) [58], whereas the percentage of individuals treated for long-term conditions such as osteoarthritis is 31% women and 23% men within the 45-64 age group and 44% women and 35% men within the 65-74 age group [2]. These findings indicate that a self-care app delivered via Facebook may have potential health benefits for a high percentage of adult users. For these reasons, we decided to use Facebook as the main platform to develop a Web-based app for self-management of knee conditions.

However, there is evidence that engagement with physical activity interventions delivered using websites tends to be low unless concerted effort is taken to address the issue [13]. Engagement can be increased by focusing on self-regulation through the use of modern devices such as mobile phones [39]. Mobile phones are globally more prevalent than computers for Internet access [59]. In addition, they are designed to be portable. For these reasons, mobile phones may provide a better platform than large screen devices (eg, desktop, laptop, or tablet) to increase engagement in the context of an exercise-based rehabilitation program. While the overall Web-based app may be too complex to be delivered efficiently on a relatively small mobile phone screen, the self-monitoring aspect of its exercise component is ideally suited for this medium. Therefore, multiple platforms are required to maximize both accessibility and engagement in a Web-based app for self-management of knee conditions. We chose to implement a Web-based intervention as a suite of apps in which a Facebook app is complemented by 2 native mobile apps.

### Needs Assessment

To ensure that the final product is optimized for its intended audience in terms of user needs, we conducted a meta-analysis of publications concerning the rehabilitation of musculoskeletal conditions to shed light on patients' information needs and self-care support requirements. Approximately 100 titles were screened and 20 of them were considered for inclusion. We conducted content analysis to group information into themes. In terms of supporting self-care, we identified the following 5 key areas: (1) sufficient and comprehensible information provision about generic and condition-specific matters; (2) tailored exercise plans that take patients' individual circumstances into account; (3) recovery monitoring based on data supplied by patients (functional test and questionnaire surveys); (4) a virtual community of patients; and (5) support for patient-doctor interactions.

### Goal Analysis

The goals defined in the needs assessment phase were broken down into component tasks by asking questions such as “In order to achieve this goal, what does the user need to know or be able to do?” Tasks were then structured around scenarios of interest and in the context of specific types of users [60]. User stories were used to document user requirements in a quick, informal way. A prioritized set of the most important user stories in our case are presented in Textbox 1.

**Prioritized set of the most important user stories.**

- As a patient, I want access to both general and condition-specific information.
- As a patient, I want to fill out questionnaires and functional tests so that I can track my progress.
- As a patient, I want to receive notifications about upcoming tests.
- As a patient, I want to see my results and compare them to other patients' averages.
- As a patient, I want visual feedback on my progress.
- As a patient, I want to know what tests I will have to do and when.
- As a patient, I want an exercise plan with detailed exercise descriptions.
- As a patient, I want to be able to talk to clinicians.
- As a patient, I want to start a new topic, post to existing ones, and comment and like other posts.
- As a patient, I want to be able to register.
- As a patient, I want to specify my demographic and medical details so that I get personalized content.
- As a patient, I want to be able to delete my account.
- As a patient, I want help on how to use the system.
- As a patient, I want a frequently asked questions (FAQs) section.

### Information Organization Scheme

An information organization scheme defines the shared characteristics of content items and influences the logical grouping of those items [61]. In the context of Web projects, it is concerned with conceptually organizing information into groups (windows, pages, tabs, and other elements of user interface) and assigning names to those elements. The organization of information is a critical success factor; if users do not understand the scheme, they will not be able to find what they are looking for regardless of how easy it is to navigate the website. We chose to implement a task-based organization scheme based on an assumption that a user will typically use the system to perform certain tasks. By extracting these tasks from user stories and grouping them together into components, we obtained a coarse-grained structural model of the required system, through which the following 5 main modules were identified: Home page, Knowledge Base, Recovery Tracker, My Self-Care Plan, and Support Group.

The Home page was envisioned to display a welcome message with some basic information about the app. The Knowledge Base was intended to serve information provision purposes. The Recovery Tracker component is meant to be an interactive tool that helps registered patients assess their recovery progress. My Self-Care Plan contains information about the self-care plan throughout typical stages of rehabilitation. In relation to My Self-Care Plan, we specifically wanted to improve accessibility to information about exercises. Therefore, we designed a mobile app to allow a user to easily access and record information about exercises when outdoors or in the gym. Finally, the Support Group should provide a venue for patients to share their experiences. The following section describes these modules and their relations in more detail.

### Implementation

**Figure 1:**
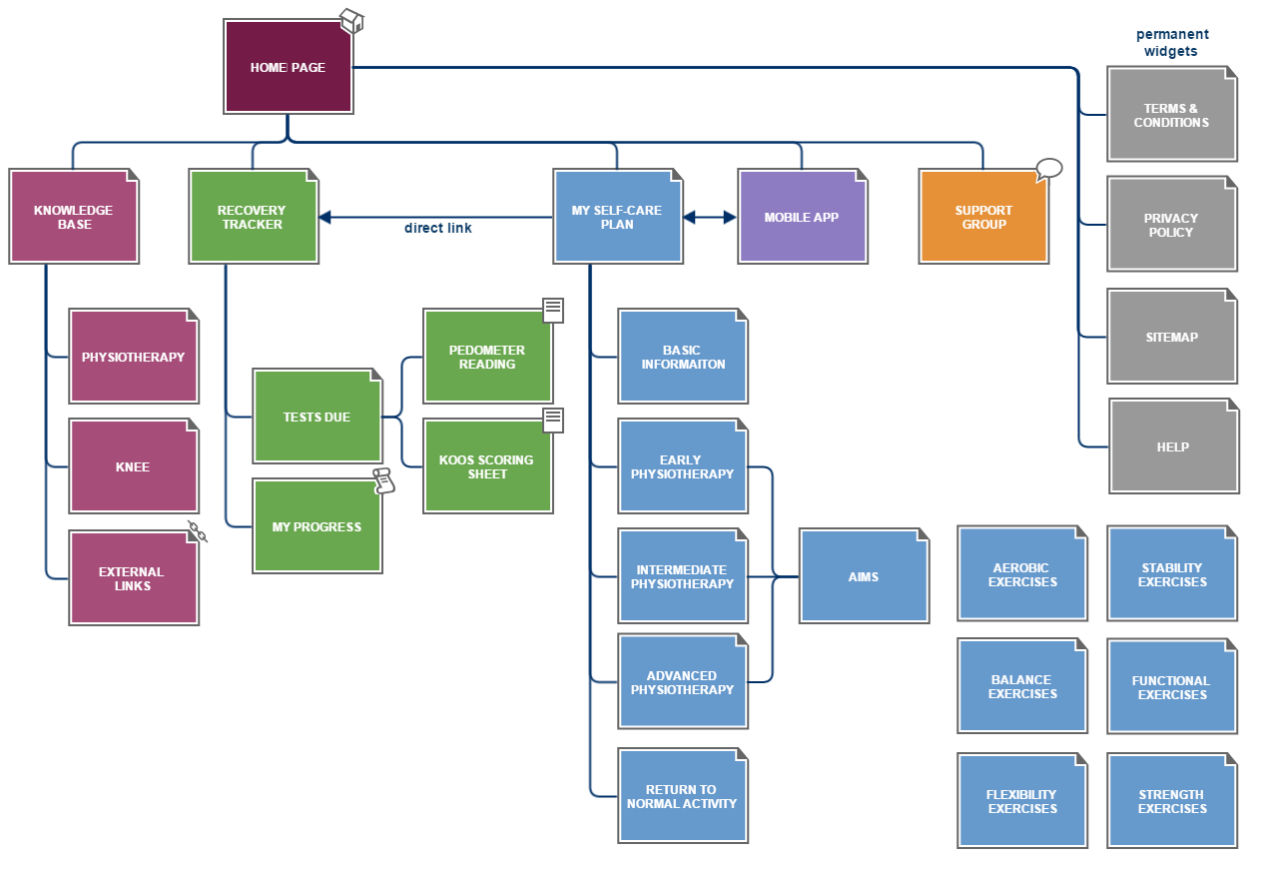
Sitemap of the Facebook app.

We developed the Taxonomy for RehAbilitation of Knee conditions (TRAK) app using extreme prototyping, a relatively new, iterative architectural approach, specifically designed for developing hypermedia apps in terms of increasingly functional prototypes [62]. Evaluation takes place at the end of each phase and prototypes are reviewed until the minimum requirements of acceptance have been met. Figure 1 shows a sitemap of the final version of the TRAK app suite. Different colors are used to distinguish between the modules whose names are listed directly under the Home page.

**Figure 2:**
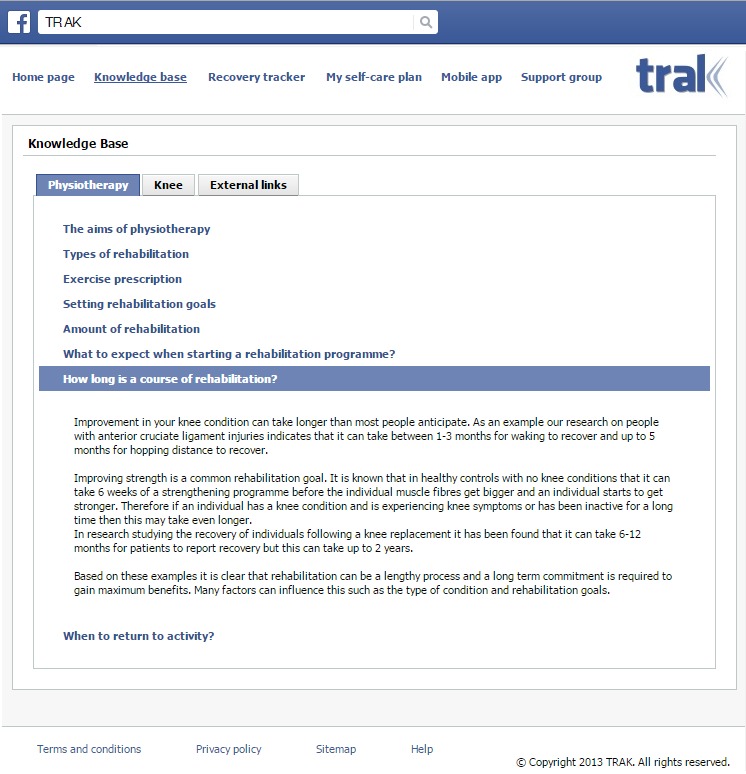
Knowledge base - Physiotherapy tab.

The Knowledge Base was intended to serve information provision purposes. Its content is grouped into 3 categories based on the results of a UK-wide survey of physiotherapists, with a specialist interest in knee conditions, regarding the types of information and advice they provide to patients [8]. The content of the 3 categories is displayed in the corresponding tabs. The “Physiotherapy” tab (Figure 2) contains articles whose purpose is to manage patients' expectations by providing information about the aims of physiotherapy, types of rehabilitation, rehabilitation goals, etc. The “Knee” tab provides information aimed at improving patients' understanding about the nature of the problem, for example, anatomy of the knee, different types of knee conditions, and the related symptoms. To get the necessary information across, our content needed to be easy to comprehend for patients with any level of health literacy and richly illustrated with graphics to reinforce understanding. Moreover, articles had to be compelling, original work, based on the best available evidence. Finally, the “External Links” tab was designed to supplement the original articles with a set of relevant links to credible external resources such as World Health Organization global recommendations on physical activity for health and Arthritis Research UK.

### Recovery Tracker

The Recovery Tracker component provides access to an interactive tool designed to help registered patients assess their recovery progress over the course of rehabilitation. It provides a schedule for collecting patient-reported outcome measures in the “Tests Due” tab, together with a checkbox to allow users to set email notification preferences. There are 2 types of patient-reported outcomes that are currently collected: subjective and objective. The subjective test is based on the Knee Injury and Osteoarthritis Outcome Score (KOOS) [63], a 42-item self-administered assessment of 5 outcomes (pain; other symptoms; activities of daily living; sport and recreation activities; and knee-related quality of life). This information is meant to help patients keep track of how they feel about their knee and how well they are able to perform their usual activities. In addition to KOOS, pedometers are commonly utilized as part of knee rehabilitation as a way of increasing physical activity levels and allowing patients to objectively assess their performance [64]. Both tests (ie, KOOS and pedometer readings) are scheduled periodically. Taking into account the complexity of the KOOS survey, and in order to allow sufficient time for significant improvement to occur, the KOOS test is scheduled monthly. Pedometers record data on a daily basis, but most are nowadays equipped with a 7-day memory function. In order for the app to be less obtrusive, the pedometer readings are collected on a weekly rather than a daily basis.

**Figure 3:**
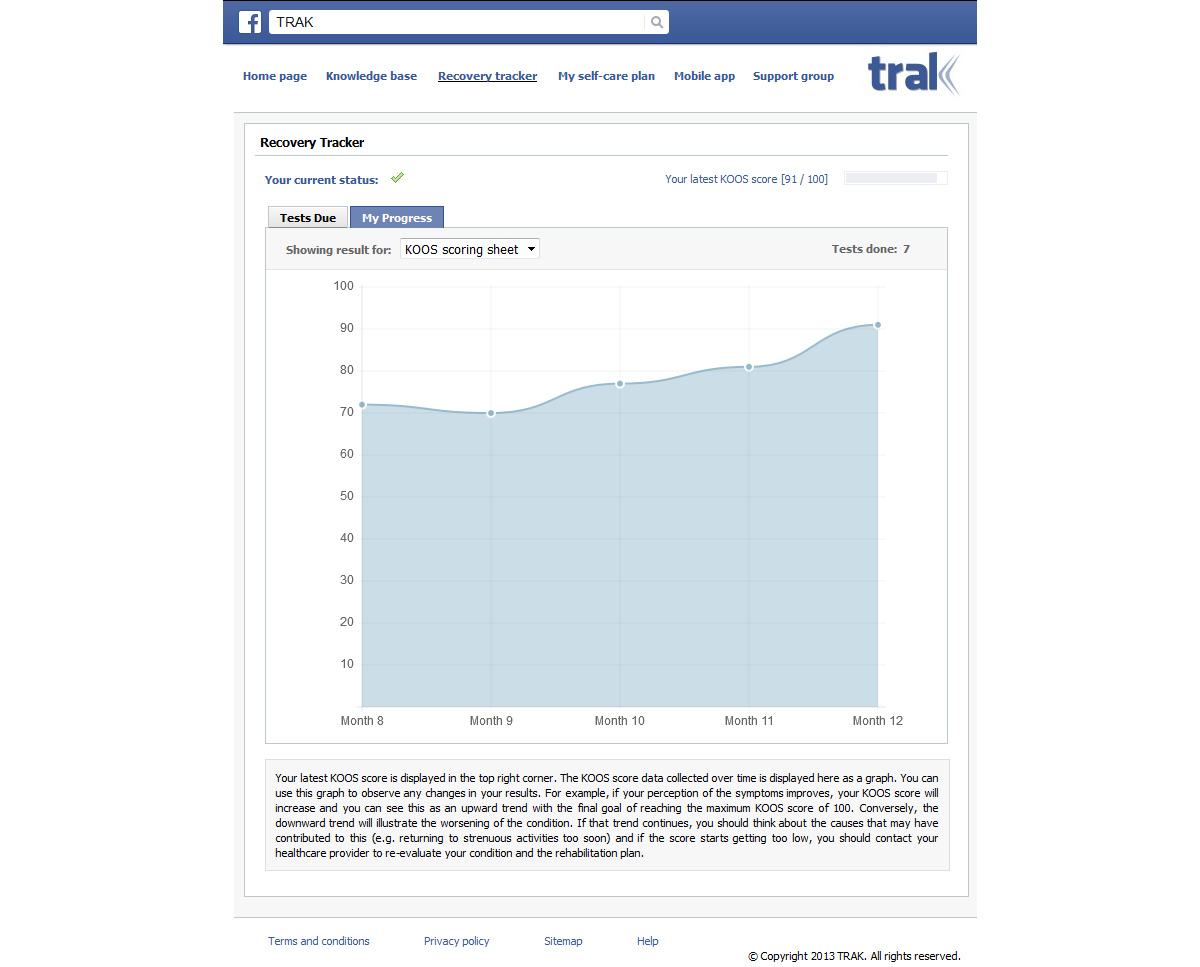
Recovery tracker - My Progress tab.

Once a patient completes a test, the system evaluates answers and computes a test score, which is compared with previous scores and the average score of other patients in the same category and stage of rehabilitation to provide the patient with feedback about their recovery. In addition to the latest test result, the patient can also track their progress over time. Namely, the “My Progress” tab provides patients with a summary of previous results (Figure 3). To simplify the interpretation of numerical information, a visual representation is provided in the form of a plot, with time expressed in weeks on the horizontal axis and test score on the vertical axis. Additional information is provided to aid interpretation of visual feedback, whose purpose is not only to inform a patient about their progress, but also to motivate them by relative improvement over time as well as to set realistic expectations by direct comparison to the average progress of other patients in similar circumstances.

### My Self-Care Plan

The overall goals of My Self-Care Plan are to help patients to control joint pain and/or swelling, regain normal knee flexion and extension, regain a normal gait pattern and stability, regain normal muscle strength, regain normal proprioception, balance and coordination for desired activities, and achieve optimal functional outcome. To support these outcomes effectively, My Self-Care Plan contains an exercise-based rehabilitation program divided into 3 phases (early, intermediate, and advanced physiotherapy) followed by the final phase (return to normal activity) [65].

**Figure 4:**
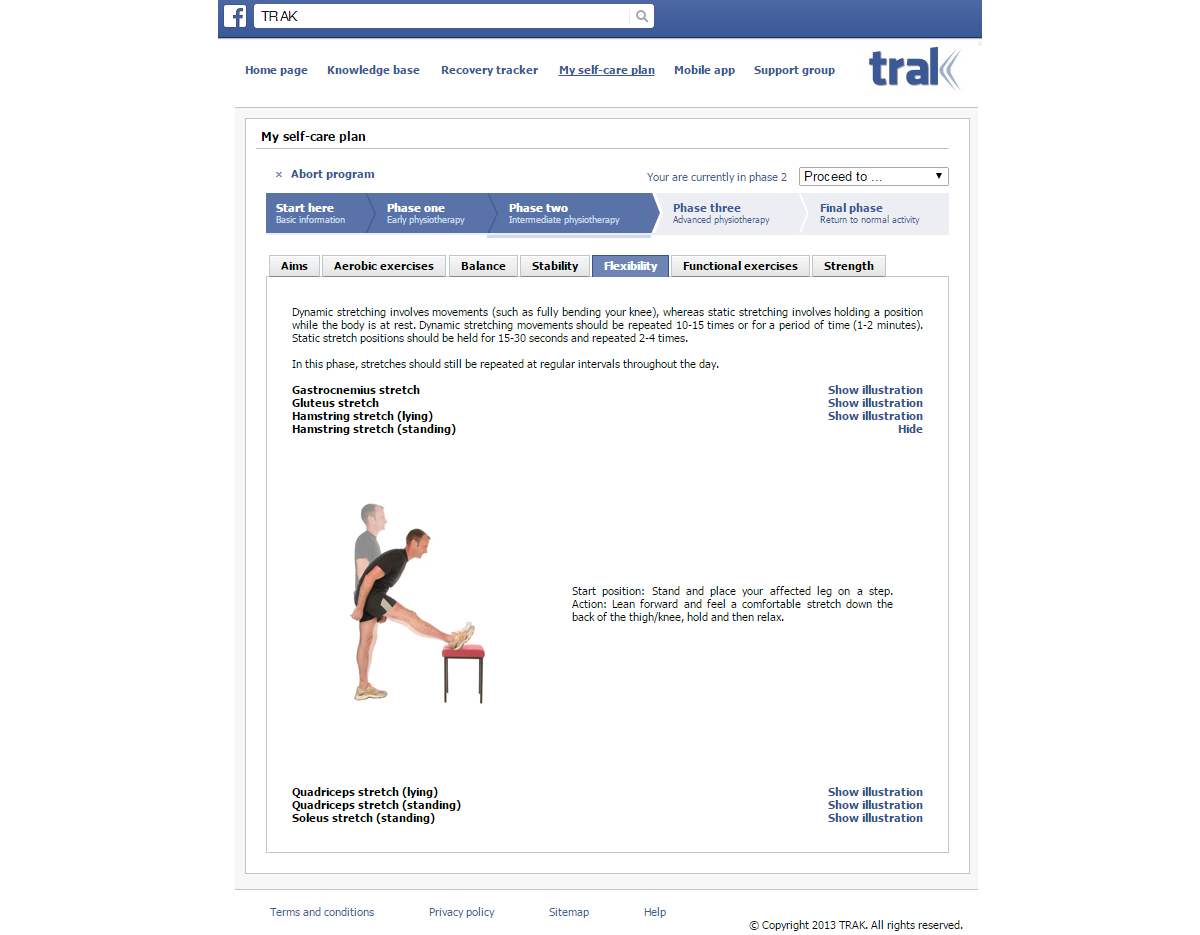
A description of a flexibility exercise recommended in Phase 2 (Intermediate physiotherapy).

The length of the rehabilitation program will depend on patients' individual circumstances, and therefore, the proposed timelines are merely a guide. Exercise progression depends on completion of the current phase, before advancing to the next one. Each phase provides information about its own aims, exercise program, and progression criteria. For example, to proceed to Phase 3 (advanced physiotherapy), patients should meet the following criteria: (1) there should ideally be no swelling of the knee, but minimal swelling is acceptable; (2) comfortable walking; (3) full range of motion; and (4) symmetrical muscle strength. In addition to these criteria, Recovery Tracker can assist patients in deciding whether they are recovering sufficiently or not. When ready to progress, the next phase of physiotherapy can be selected from a drop-down menu (Figure 4). By default, the exercises shown when selecting My Self-Care Plan belong to the current phase.

**Figure 5:**
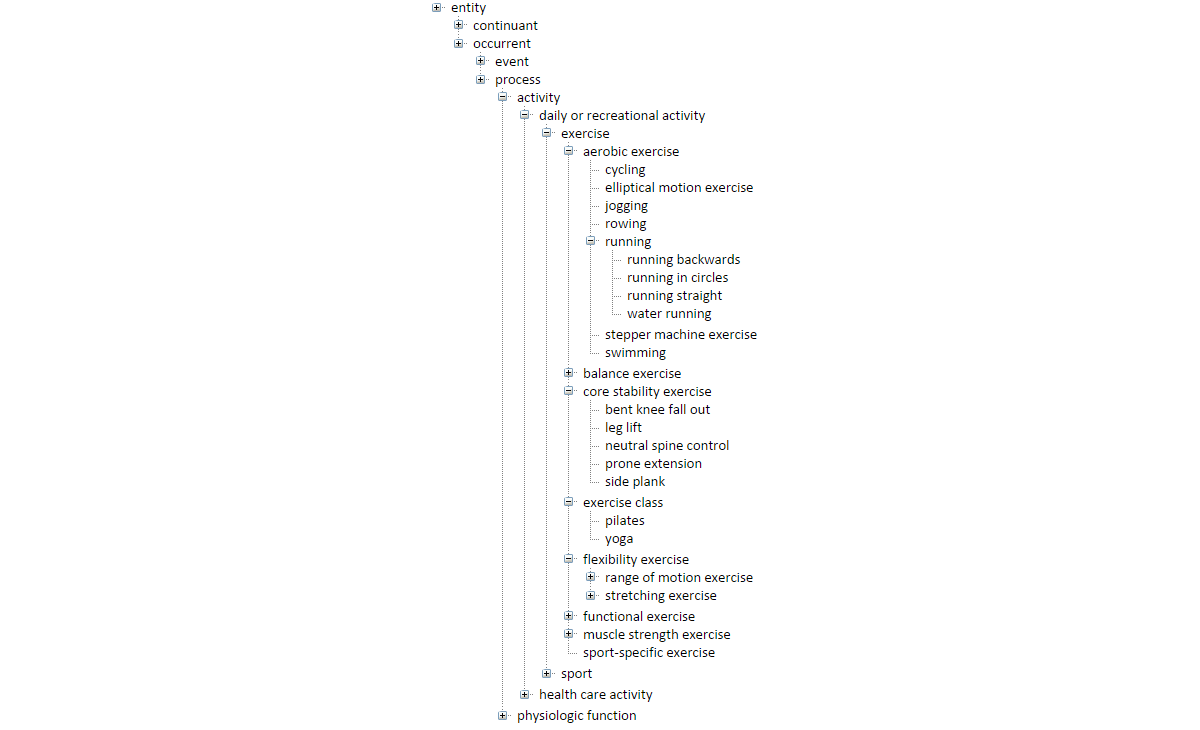
Partially expanded TRAK ontology provides the classification of exercises.

Each rehabilitation phase is supplemented with information about its aims, advice, and a reference to Recovery Tracker. The exercises associated with each phase are divided into 6 groups, namely, aerobic, balance, stability, flexibility, functional, and strength exercises, which are accessible via the corresponding tabs. Each tab is populated with specific exercises appropriate for the given phase. Each exercise comes with a collapsible/expandable illustration section, which contains an image and a short description (Figure 4). The exercise information originates from the TRAK ontology, which has been specifically designed to formally model standard care for the rehabilitation of knee conditions [8]. It incorporates over 100 exercises organized hierarchically (Figure 5). These exercises were selected through a systematic literature review [7] and a UK-wide survey of clinical practice. The TRAK ontology is encoded in the OBO flat file format, version 1 [66], which makes it machine readable and thus reusable in a variety of informatics apps. In other words, the content of the TRAK ontology can be automatically imported where needed. Textbox 2 provides an example of an exercise represented in the ontology. Such representation provides a unique identifier for each exercise, its preferred name, together with any other synonyms, definition, cross-references to external sources (eg, UMLS), classification using “is-a” relationship, and named relationships to other relevant concepts in the ontology.

**An example of exercise description in the TRAK ontology.**

[Term]

id: TRAK:0000405

name: backward lunges

def: "Start position: Stand upright. Action: Step backwards with the affected leg and bend the affected knee until it is flexed 90 degrees, then slowly straighten up keeping the body weight on the affected leg and step back with the unaffected leg." []

synonym: "reverse lunges" EXACT []

xref: UMLS_CUI:C2019591

is_a: TRAK:0000135 ! lunges

relationship: performs TRAK:0000157 ! concentric contraction

relationship: performs TRAK:0000158 ! eccentric contraction

**Figure 6:**
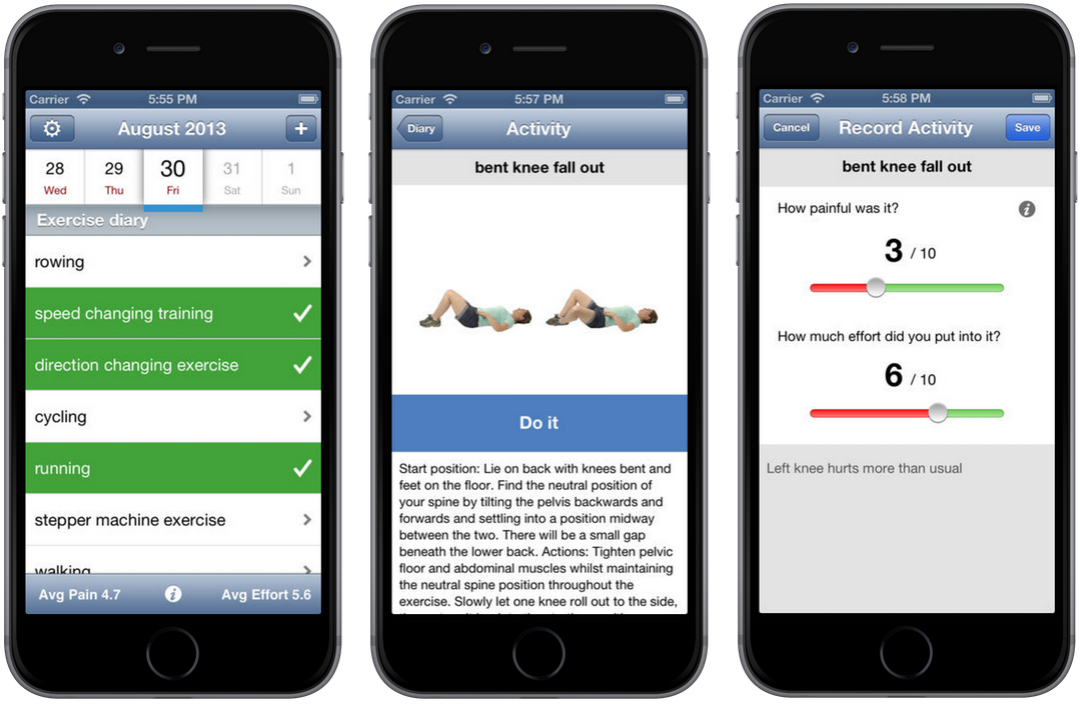
TRAK mobile app allows a patient to keep track of exercises on the go.

In relation to My Self-Care Plan, we specifically wanted to improve accessibility to information about exercises in order to better engage patients in the self-care program. Therefore, we designed a native mobile app to allow a user to easily access and record information about exercises without having to rely on an Internet connection, which may not be available in typical exercise environments, for example, outdoors or in the gym. Namely, My Self-Care Plan provides a selection of appropriate exercises as the patients progress through the 3 rehabilitation phases, while the mobile app allows them to keep a diary of exercise activities as they actually do them at home or somewhere else. The mobile app's functionality includes exercise selection, access to exercise instructions with an image, logging an exercise together with pain and effort required for its completion as well as any other comments, and tracking progress by monitoring pain and effort over time (Figure 6). As before, all exercise-related information was reused from the TRAK ontology. Any changes to the ontology stored on a cloud server are propagated automatically, thus allowing the mobile app to evolve seamlessly.

### Virtual Support Group

The evidence suggests that the capacity of patients to self-manage can be sustained through the perceived social support [67]. In particular, social support proved to be an important external factor influencing how much participants exercise [68]. Therefore, we set up the Support Group to provide a venue for patients to share their experiences. It has been implemented as a Facebook group rather than as a discussion forum in order for the Support Group to be more interactive and familiar to the intended user group. Indeed, Facebook groups provide a platform that seamlessly supports communication, sharing, and interaction within a social group gathered around individuals with common interests [69]. Facebook groups come with intuitive privacy settings to control who can see group's membership and content: secret (only members can find the group and see posts), closed (anyone can find the group and see its members, but only members can see posts), and open (anyone can see the group, its members and their posts). To create a private social space for patients, we opted for a secret group. Because the group cannot be found using Facebook's search function, new members can be added by invitation only. Privacy concerns are addressed through policy and education. In terms and conditions, members are reminded that confidentiality of discussions is expected, but cannot be guaranteed. As with any online communication, anything posted within the group has the potential to become public, thus patients are warned to only post information they are comfortable sharing with others.

###  Measures

Following the initial app development, its comprehensive testing, and research ethics committee approval, a usability study was conducted to evaluate the usability of the TRAK app suite. There were 3 types of participants that were recruited for the study: software experts, patients, and physiotherapists.

### Participants

A total of 44 participants (29 patients and 15 physiotherapists) were recruited through the Physiotherapy Department at the Cardiff and Vale University Health Board. Eligibility criteria were (1) an ongoing knee condition for patients and specialist interest in knee rehabilitation for physiotherapist, (2) aged 18 years or older, as we targeted adult users, and (3) having used a Facebook account at least once a week for more than a month. Potential participants were excluded if they (1) had no Internet access at home, (2) did not speak the English language with native-like proficiency, or (3) had contraindications for physical activity without medical supervision. All participants signed the informed consent document. No incentives were offered for participation in the study.

Prior to approaching patients and physiotherapists, 3 software experts were recruited to provide initial feedback on usability. They were asked to register as users on the app and complete a set of tasks to provide an informed opinion about potential usability issues and suggest possible improvements. They provided written feedback, which was then analyzed to identify the following usability themes: design, feedback, format, instructions, navigation, terminology, and learnability. These themes provided the basis for the development of the usability questionnaires, which were used to collect both qualitative and quantitative information.

### Questionnaires

The questionnaires were divided into 3 parts: (1) Questionnaire 1: computer and Internet usage; (2) Questionnaire 2: task completion; and (3) Questionnaire 3: subjective user preferences.

All 3 questionnaires were accessible online. Patients were recruited during an appointment at the clinic and were instructed to complete Questionnaire 1 at home and use the app as part of their self-care. They completed Questionnaires 2 and 3 at the clinic during the follow-up appointment. Of the 29 patients, 13 did not actively participate in the study. Of the 16 remaining patients, only 1 did not complete Questionnaire 1, but they all completed both app-specific Questionnaires 2 and 3. The responses of all 16 patients were used to provide the summary reported here. Of the 16 patients, 10 were men and 6 were women, whose age ranges were 22.83 ± 4.36 years and 23.60 ± 3.24 years, respectively. As for the physiotherapists, all 15 participants completed all 3 questionnaires. Of the 15 physiotherapists, 11 were women and 4 were men, whose age ranges were 36.45 ± 4.59 years and 40.25 ± 4.57 years, respectively.

It is argued that 5 participants would reveal 80% of usability problems [70,71]. Therefore, a total of 34 (3 + 16 + 15) participants seem sufficient in the context of this usability study. In particular, we have at least five participants from both types of stakeholders: patients and physiotherapists. Their total numbers (16 and 15, respectively) are also evenly balanced, which should provide a similar level of insight into their views on usability.

## Results

### Questionnaire 1: Computer and Internet Usage

**Figure 7:**
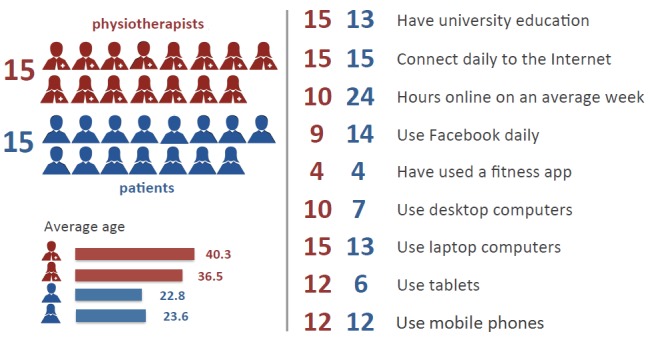
A summary of responses about computer and Internet usage.

Questionnaire 1 consisted of 19 questions whose main purpose was to verify the computer literacy of the participants as part of the inclusion criteria, but also to try and relate the response to task-based questions to different levels of computer literacy. Figure 7 shows a summary of responses to Questionnaire 1.

Of 15 patients who completed Questionnaire 1, all had at least secondary education out of which 13 had a university degree. All of them were accessing the Internet daily (on average 24.2 hours per week) at home mostly via a laptop (87%, 13/15). All 15 patients were mostly using the Internet to send or read email and access an SNS. Of the 4 SNSs (Facebook, Twitter, LinkedIn, and Google Plus), Facebook was the only site regularly used by all participants, followed by Twitter, which was used by 9 of 15 patients. LinkedIn was never used by 13 of the patients, and Google Plus was never used by 12 of the patients. With the exception of 2 patients, all others were likely to use Facebook on a typical day. They used Facebook mostly to communicate with friends and access content on Facebook pages, and somewhat less often to participate in Facebook groups. Almost half of the patients never used Facebook apps prior to this study. Laptops and mobile phones were the most used devices to access Facebook, with laptop being the preferred choice. Most patients (73%, 11/15) have never used an app such as Weight Watchers or iFitness to get in shape. The 4 patients who have used such apps liked their mobile aspect and the ability to use them on-the-go to track exercise performance, but they disliked crashes, frequent updates, large downloads, cost, and some of the apps not being available on Android devices.

Of the 15 physiotherapists, all held a university degree. All of them were accessing the Internet daily (on average 10 hours/week) at both home and work mostly via a laptop (100%, 15/15). All 15 physiotherapists were mostly using the Internet to send or read email and search for information online. Of the 4 SNSs (Facebook, Twitter, LinkedIn, and Google Plus), Facebook was used by 14 physiotherapists, 12 of whom used it on a regular basis (ie, weekly or daily). However, in contrast to patients, only 5 physiotherapists were likely to use Facebook on a typical day. They used Facebook mostly to communicate with friends and access content on Facebook pages, and less often to participate in Facebook groups. A total of 11 physiotherapists never used Facebook apps prior to this study. Laptops, tablets, and mobile phones were the most used devices to access Facebook, with tablets being the preferred choice. Like patients, a majority of physiotherapists (73%, 11/15) have never used an app to get in shape. The 4 physiotherapists who have used such apps liked their ability to demonstrate exercises and track performance, but in addition to frequent updates, large downloads, and cost, they also disliked their effect on battery life as well as some not being user friendly.

### Questionnaire 2: Task Completion

As part of completing Questionnaire 3, participants were asked to complete a series of prespecified tasks using the app. A total of 15 tasks were defined in order to test the usability of all aspects of the app. The behavior of each user was studied in an individual session. Screencasting software [72] was used to record activities on the computer screen during the task-based sessions. The videos were analyzed to explain any difficulties that lead to incorrect answers to task-based questions.

A total of 16 patients completed Questionnaire 2, most of whom successfully completed the tasks associated with the questions. A total of 10 patients (62%, 10/16) successfully navigated to the Knowledge Base and correctly identified the number of knee conditions described. The remaining 6 patients incorrectly identified the number of knee conditions as 3, which is the number of subheadings under the Knee Conditions tab: Anatomy, Knee Symptoms, and Knee Conditions. This feedback was later used to rename the tab to a more appropriate and less confusing title—“Knee.” A total of 12 patients (75%, 12/16) successfully accessed information via External Links provided in the Knowledge Base. All but 1 patient was able to access the training video on YouTube.

A majority of patients (62%, 10/16) were not able to correctly identify the connections on the Sitemap, either because they were not able to interpret the Sitemap diagram or because they misinterpreted the question and ignored the Sitemap completely. The remaining 6 patients correctly identified that the Mobile App module was only directly linked to My Self-Care Plan. To rectify this, we added a reference to Sitemap to the Home page and explained that its purpose is to illustrate the structure of the app through the use of pages, tabs, and link. Further, all patients were able to identify the currently selected module, report their latest KOOS, and check when their next pedometer reading was due for submission.

A total of 10 patients (62%, 10/16) successfully navigated to the iTunes page from which the mobile app was available for download. The remaining 6 patients failed to scroll down to be able to see a link to iTunes. For this reason, we moved the link higher up, as it was not visible on the laptop screen used during the task-based sessions. Most patients (75%, 12/16) did not own an iOS-compatible device, and, therefore, were not able to perform the tasks using the mobile app. In response, we implemented an Android version of the mobile app.

A majority of patients (81%, 13/16) successfully identified the number of exercises described in Phase 3 of My Self-Care Plan. The remaining 3 patients counted the number of strength exercises in Phase 1 instead. Most patients (88%, 14/16) were able to correctly interpret the description of a specific exercise, but 2 failed to correctly describe it as they seemed to ignore the explicit textual description, which stated "Start position: Stand upright. Action: Lift the unaffected leg...." For example, one patient stated the start position as "stand on left leg" by literally interpreting the image provided. The other one misinterpreted the description as "affected knee off floor...."

All but 1 patient successfully identified the privacy settings in the Support Group. However, most patients (81%, 13/16) were not aware of the types of personal information stored by the app even though it is clearly specified in the privacy policy. To direct users to this information, we added a reference to the Privacy Policy on the Home page. We also added an explanation on how to delete an account, together with the associated information.

A total of 15 physiotherapists completed Questionnaire 2, most of whom successfully completed the tasks associated with the questions. Only 1 physiotherapist incorrectly identified the number of knee conditions as 3, which was again attributed to an ambiguity in naming the tab under which the subheading “Knee Conditions” is located. As indicated earlier, the tab in question was subsequently renamed to better reflect its content. A total of 11 physiotherapists (73%, 11/15) successfully accessed information via External Links provided in the Knowledge Base. A total of 8 physiotherapists were able to access the training video on YouTube, but the remaining 7 have not attempted to look for the training video.

A slight majority of physiotherapists (53%, 8/15) were able to correctly identify the connections on the Sitemap; 4 other physiotherapists misunderstood the questions, but 3 correctly provided the total number of connections. The remaining 3 physiotherapists misinterpreted the question and ignored the Sitemap completely, thus not providing a correct response. We explained the corrective action taken previously in response to patients' feedback. Like patients, all physiotherapists were able to identify the currently selected module, report their latest KOOS, and check when their next pedometer reading was due for submission.

All but 1 physiotherapist successfully navigated to the iTunes page from which the mobile app was available for download. As indicated earlier, we moved the link higher up to make it visible without the need to scroll down. While most patients (75%, 12/16) did not own an iOS-compatible device, a majority of physiotherapists (67%, 10/15) did own one, which allowed us to get more insight into the usability of the mobile app. All of them were able to select an exercise and record information such as pain level. Only 1 physiotherapist did not manage to find previously recorded information.

A majority of physiotherapists (93%, 14/15) successfully identified the number of exercises described in Phase 3 of My Self-Care Plan. There was 1 physiotherapist who incorrectly entered the number of exercises as 6, but the analysis of the video showed them pointing at the 5 exercises provided, so we assume that this was a typographical error. Partly relying on their specialist knowledge, all physiotherapists were able to correctly interpret the description of a specific exercise.

All but 1 physiotherapist successfully identified the privacy settings in the Support Group. Still, like patients, the vast majority of 12 physiotherapists were not aware of what types of personal information were stored in the app. As indicated before, we made an explicit reference to the Privacy Policy on the Home page with an explanation on how to delete information from the app.

### Questionnaire 3: Subjective User Preferences

Questionnaire 3 was designed to highlight subjective user preferences about the app including its general usability, user perception, and appropriateness in the context of exercise-based rehabilitation. The part about general usability was based on the System Usability Scale (SUS), a questionnaire for assessing the perceived usability of interactive systems [73]. It consists of 10 questions based on a 5-point Likert scale (1=strongly disagree, 5=strongly agree). In comparison to other commonly used questionnaires, it was shown to be the simplest and most reliable in determining website usability [74]. Not surprisingly, it is the most used questionnaire for measuring perception of usability. The overall SUS score is calculated on a scale from 0 to 100. The widespread usage of the SUS questionnaire allows the usability of a system to be benchmarked against others. Based on the average SUS score, any score above 68 is considered above average. In our case, the SUS score calculated from patients' responses was 78, whereas that of physiotherapists was 75, which belong to a percentile range of 80-84% and 70-79%, respectively. In other words, our app has higher perceived usability than 70% of systems. This rank can be interpreted as “Grade B” on a scale from A to F [75].

**Figure 8:**
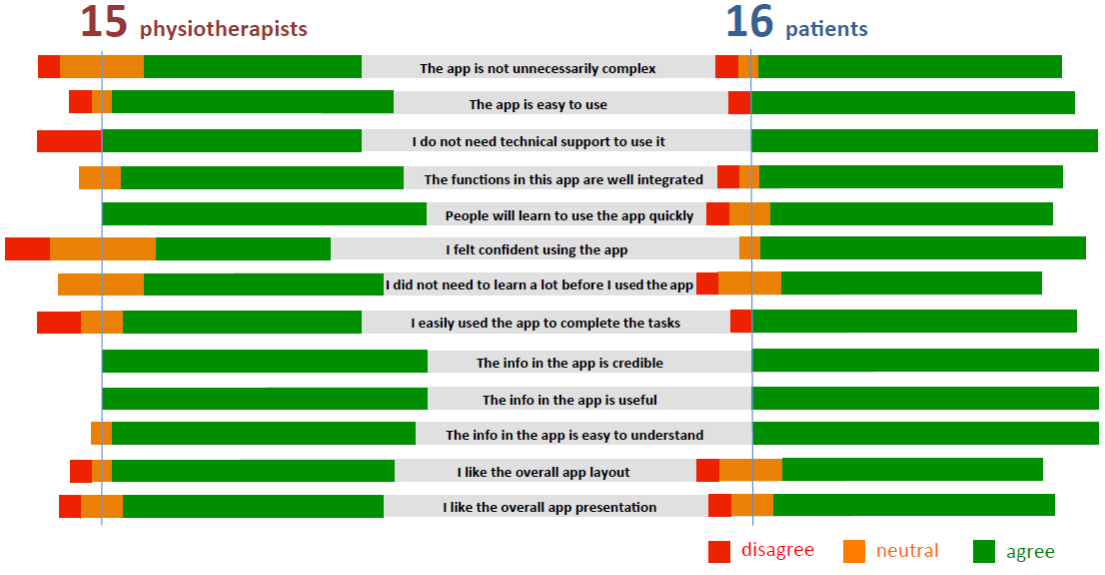
A summary of responses about user perception.

Figure 8 shows a summary of responses about user perception. Only 1 patient out of 16 found the app unnecessarily complex, cumbersome, inconsistent, difficult to learn, not easy to use, and not well integrated. None thought they would need technical support to use the app. Similarly, a single physiotherapist out of 15 found the app unnecessarily complex and not easy to use. There were 3 other physiotherapists that thought that they would need the support of a technical person to be able to use the app. They also did not feel confident using the app. By contrast, various functions in the app were found to be well integrated with no inconsistencies. All physiotherapists agreed that most people would learn to use the app very quickly. It seems that the physiotherapists experienced more difficulties in using the app than the patients, which was reflected in a slightly lower SUS score.

In addition to SUS-based questions on general usability, we formulated 8 additional diagnostic questions. All patients agreed that the information provided was credible, useful, and easy to understand. The physiotherapists agreed with patients' views that the information provided was credible, useful, and easy to understand. However, 1 physiotherapist disliked the layout of the app, and another disliked the presentation of the app.

Finally, 14 questions were asked in relation to the appropriateness of the app in the context of exercise-based rehabilitation. The patients unanimously agreed that exercise descriptions were sufficiently clear and that the images matched the description well and made it easier to follow the instructions. However, 12 of 16 patients (75%) said that they would prefer videos to images. Most patients (69%, 11/16) felt encouraged to exercise more. With the exception of only 2 patients, all others felt able to progress the exercises using the app and that it improved the self-management of their knee condition. A slight majority of patients (56%, 9/16) believed that using the app facilitated the recovery from knee condition. A total of 14 patients (88%, 14/16) would recommend the app to a friend, colleague, or family member who suffered from a knee condition. Only 1 patient was concerned about the privacy or security regarding the app's use.

When asked about an optimal number of exercises that should be available in the app, most physiotherapists explicitly stated that the current content was appropriate in terms of quantity and variety. With the exception of a single physiotherapist, the rest unanimously agreed that exercise descriptions were sufficiently clear, and that the images matched the description well and made it easier to follow the instructions. Similarly to patients, 11/15 physiotherapists (73%) said that they would prefer videos to images. In line with the patients' views, the majority of physiotherapists (67%, 10/15) believed that patients would be encouraged to exercise more. With the exception of 1 physiotherapist, the professional opinion of the remaining 12 physiotherapists (80%, 12/15) was that the information provided was sufficient to be able to progress the exercises. More physiotherapists (11/15) than patients (9/16) believed that using the app could facilitate the recovery from a knee condition. Patients were seen 2 weeks following the recruitment, which is a relatively short period for patients to observe a significant recovery, but obviously the prevalent professional opinion based on clinical practice was that such recovery would be noticeable over time. The same subset of 11 physiotherapists agreed that using the app could improve the self-management of a knee condition. All 15 physiotherapists would recommend the app to someone who suffered from a knee condition. Whereas only 1 patient was concerned about the privacy or security regarding the app use, 4 physiotherapists expressed such concerns.

We analyzed patients' opinions on whether the app could support face-to-face appointments with a physiotherapist and extracted 6 main themes given in Table 1. Likewise we also analyzed physiotherapists' opinions (Table 2), which revealed the overall agreement on the given issues from 2 different perspectives.

**Table 1:** A sample of patients' opinions on the extent to what the app could support face-to-face appointments.

Theme	Example
Facilitate communication	It could form a good trigger for talking points or examples; for example, I tried the third knee stability exercise and really struggled with it.
Improve understanding	If unsure of any exercise, I can easily access the app to clear any confusion.
Provide information	It gives extra exercises I can use on top of what my physiotherapist has given me.
Support self-management	Providing a head start to complete the tedious exercise on your own.
Enable progression	It would be a good reminder of the exercises explained by your physiotherapist and how to do them, and encourage you how to progress them if they become too easy.
Recall information	It can be a good reminder of the exercises the patient is supposed to do. I have often come away from a physiotherapy appointment with so many exercises swimming round in my brain that I get them all a bit mixed up.

**Table 2:** A sample of physiotherapists' opinions on the extent to what the app could support face-to-face appointments.

Theme	Example
Facilitate communication	This app would streamline discussions to relevant areas, saving the clinician's (and patient's) time.
Improve understanding	Improve ability to check exercises against a clear picture and description.
Provide information	The quality and accessibility of information are much better than any that we currently provide at face-to-face rehabilitation sessions.
Support self-management	I think this is a great resource, which can back up and add to the information provided by therapists and allow patients to be much more in control of their own rehabilitation strategies.
Enable progression	Physiotherapist can also highlight exercises to progress onto knowing the patient will have a clear instruction at the appropriate time.
Recall information	Good as a reminder of exercise technique and to stop the need to draw stick men exercises.

###  Face-to-Face Appointments and the App

Further, we analyzed the patients' opinions on whether the app could replace face-to-face appointments with a physiotherapist. The overwhelming majority of patients (n=10) felt strongly that while the app would be a useful aid to their rehabilitation, it could by no means substitute face-to-face contact. The main reasons they provided were related to the reassurance associated with human contact as well as the potential to provide personalized care. However, 3 patients believed that, while the app could not completely replace face-to-face appointments, it could certainly reduce the number of such appointment. There were 2 patients who found this aspect particularly useful, quoting logistic reasons such as time constraints and transport links, which at times make it difficult for them to attend appointments. This implies that the app would be of high value to patients with restricted access to health care premises, for example, because of impaired mobility, remote locations, and/or nonflexible working conditions.

Not surprisingly, the physiotherapists correctly predicted that patients would not be keen on fully replacing face-to-face appointments. However, they themselves were more open to the idea of reducing the number of face-to-face appointments (10 of 15 physiotherapists), typically suggesting alternative forms of contact such as telephone or Skype conversations, but also embedding a direct contact into the app itself. In contrast to patients who specified reassurance as the main reason for insisting on face-to-face appointments, physiotherapists named monitoring and progression as their professional reasons.

Finally, when asked about any other comments, 5 patients provided no additional comments, 8 patients were extremely positive about the app (Table 3), and 3 left very specific comments about particular aspects of the app (eg, number of clicks, amount of information). A total of 11 physiotherapists shared the overwhelmingly positive reaction to the app (Table 4). There were 3 physiotherapists who left specific comments about the ways of improving the app (eg, adding contact option, measuring adherence).

**Table 3:** A sample of patients' comments.

Theme	Example
Uptake	The TRAK app is well set out and it's easy to access different areas. The information provided is thorough and clears any confusion about any knee problems. I will indefinitely be using it as of today!
Progression	Great app in general and very helpful, exactly what's needed to securely help patients along the road to recovery.
Content	I thought the app was exceptional, really interactive and very clever. My only comments would be for the information surrounding the injury/knee joint (medical bit) could be slightly shorter worded and perhaps be written in bullet-point form.
Technology	I rarely used the Facebook app, was barely aware Facebook did apps. If the full (Facebook version) of this app was available for iPhone with the instructions, plan etc, I would have used it a lot more and found it far more useful.

**Table 4:** A sample of physiotherapists' comments.

Theme	Example
Uptake	May be useful to add a goal setting function/monitor progress with specific functional/fitness goals. Overall, think it is an excellent idea and would definitely consider using it with patients, clearly a lot of work gone into developing the app/information & exercises. I look forward to becoming more familiar with it and using it more.
Progression	Looks really good. Would be great to build a database to see how a cohort of knee conditions progress through their rehab and return to function/work/sport. Physiotherapists could use this information to spot those failing to progress earlier, and help inform patients more accurately about their prognosis/timescales.
Content	Good. I think videos of patients doing the exercises in a good or bad way would be even greater.
Technology	Great idea for younger/tech savvy patients who are self motivated. Would there be a pop up reminder regarding follow up appointments in physiotherapy? Might be useful if appropriate.

### App Feedback

In conclusion, the results of the usability study informed the subsequent improvements of the app by considering all aspects that have an impact on user experience. This section outlined a set of measures taken in response to issues raised in user feedback. Minor changes included renaming and rearranging the content of the app to improve navigation, content, and facilities designed to help users' learning experience.

A major change was the addition of an Android app to the app suite. This issue was first identified in patients' response to Questionnaire 1 (computer and Internet usage), when patients who had prior experience with using fitness apps raised an issue of some of these apps not being available on Android devices. The need for an Android version of the mobile app became obvious when the responses in Questionnaire 2 (task completion) highlighted that the majority of patients (75%, 12/16) did not own an iOS-compatible device. Indeed, these results are in line with data reported by the International Data Corporation [76], a global provider of market intelligence for the information technology, telecommunications, and consumer technology markets. According to their Worldwide Quarterly Mobile Phone Tracker, Android dominates the mobile phone operating system market share, accounting for nearly 85% in comparison to iOS, which accounts for only 12%. Android's market share has been increasing steadily over the last 4 years from 36%, 70%, 80%, to 85% during the period from 2011 to 2014. Obviously, Android is by far the most popular operating system on mobile phones. Therefore, ignoring this platform would alienate a vast majority of potential users of the TRAK app suite. The Android version of the mobile app was implemented following the conclusions of the usability study. We incorporated information about it into the Mobile App module of the Facebook app. The 2 mobile apps are equivalent in their functionality and both can be used as standalone products.

##  Discussion

### Limitations of the Study

This paper described a novel Web-based app for delivering exercise to patients undergoing knee rehabilitation and provided preliminary usability findings. A limitation of the usability study was the discrepancy between the age range (19-31 years) of the patient sample and the fact that the risk of developing osteoarthritis increases from the late 40s [2]. Potential usability issues related to the app design in the context of the physical abilities and the visual capacity of older users can be resolved relatively easily. A major concern, however, is that of digital literacy across the age groups. Not surprisingly, Internet usage was found to fall with age across Europe, but the increasing development of useable and accessible products such as mobile phones and mobile apps is expected to reduce the challenge of digital literacy [77]. In Europe, the Riga Declaration 2006 [78] established specific targets in relation to aging and information and communication technologies, one of which is to halve the gap in average Internet use between older people and the EU population. In this context, the usability results can be generalized beyond the age range of the patient sample used in the study.

The usability study uncovered various areas of possible improvements. Both patients and physiotherapists suggested that videos should replace images as illustrations of exercises. We already produced over 20 exercise videos. From a developer's perspective, replacing images with videos would constitute only a minor change to the system. Although quoting different reasons, all stakeholders expressed a wish for the app to close the feedback loop between patients and physiotherapists by allowing them to exchange information and communicate directly as part of a shared decision-making process. Indeed, support for patient-doctor interactions was identified during needs assessment. Incorporating a Web chat function [79] into the app would allow a patient to chat with a physiotherapist in real time. In technical terms, it would simply require a developer to reuse a piece of ready-made code. The reason we did not implement this functionality was due to human resource constraints. While it would be worth investing in this particular aspect of a Web-based intervention, the limitations on human resources are likely to persist. A fully automated question-answering system (QAS) or at least a list of FAQs would provide a compromise solution to this problem. As part of piloting the Web intervention within the NHS, plans are already in place to collect FAQs and incorporate them into the TRAK app suite. In combination with the TRAK ontology, the collected questions and answers will provide a basis for further research into developing a QAS.

Another possibility to improve the function of the support group would be to involve a health care professional as a moderator. Namely, such groups provide a communication space, but not a self-sustaining conversation. A moderator strategy is required to support community development. There are 2 ways of moderation that are essential for health promotion interventions: (1) professional supervision to maintain a safe space for discussion and information quality, and (2) a more engaged presence to improve activity and timely response to user requests [80].

Future work will support the professional needs of physiotherapists by allowing them to specify exercise prescription within the app as part of personalizing its exercise program according to individual patients' circumstances. The TRAK ontology will drive the exercise selection process. The ontology will also facilitate tighter integration of the individual apps within the TRAK suite. In particular, we want to exemplify the correlation between exercise adherence (currently monitored by the mobile app) and recovery progression (currently quantified by the KOOS within the Facebook app). In addition to providing feedback and motivation to patients, such information would allow physiotherapists to monitor patients' recovery and exercise progression. On a large scale, these data can support epidemiological studies to identify the most effective treatment components, so that new interventions can be developed.

The assessment of long-term impact of a Web-based intervention on knee rehabilitation was outside the scope of this study. Nevertheless, our work laid out the foundation for further translational research based on a randomized control trial. We recently acquired funding to put the TRAK intervention into practice within the Cardiff and Vale University Health Board and gather evidence about how such innovation improves quality of health care. This will provide an opportunity to explore the link between face-to-face physiotherapy interaction and the use of the app in light of the finding that better outcomes in Web-based interventions were identified when there were multiple contacts with participants and when the time to follow-up was short [14].

### Conclusions

The aim of this study was to assess the usability and acceptability of a Web-based intervention in knee patients and physiotherapists who deliver knee rehabilitation. We developed TRAK, a Web-based app suite, to support self-management of knee conditions. Its content is based on the TRAK ontology, which includes rehabilitation concepts and treatment modalities that are part of standard care for the rehabilitation of knee conditions based on expert clinical opinion and published research evidence [8]. The usability study suggested unanimous acceptability by both types of stakeholders. Both patients and physiotherapists agreed that the given Web-based approach would facilitate communication, provide information, help recall information, improve understanding, enable exercise progression, and support self-management in general. The Web app was found to be easy to use and user satisfaction was very high. These results suggest that a Web-based intervention is feasible and acceptable in supporting self-management of knee conditions.

## Abbreviations

FAQ: frequently asked questions
KOOS: Knee injury and Osteoarthritis Outcome Score
NHS: National Health Service
QAS: question-answering system
SNS: social networking site
SUS: System Usability Scale
TRAK: Taxonomy for RehAbilitation of Knee conditions
